# Editorial: Nutrition and sustainable development goal 4: quality education

**DOI:** 10.3389/fnut.2024.1447032

**Published:** 2024-06-26

**Authors:** Sarah Burkhart, Clinton Beckford, Elezebeth Mathews, Alemneh Kabeta Daba, Abul Hasnat Milton

**Affiliations:** ^1^Australian Centre for Pacific Islands Research and School of Health, University of the Sunshine Coast, Maroochydore, QLD, Australia; ^2^University of Windsor, Windsor, ON, Canada; ^3^Department of Public Health and Community Medicine, Central University of Kerala, Kasaragod, Kerala, India; ^4^College of Medicine and Health Sciences, Hawassa University, Hawassa, Ethiopia; ^5^Research and Training International, Newcastle, NSW, Australia

**Keywords:** school, nutrition, food, sustainability, education—teaching and evaluation

Nutrition sits at the center of the sustainable development goals (SDGs) because of its multisectoral nature and multidimensional contribution. In addition to achieving “Zero Hunger” (SDG2), improvements in nutrition are critical to both achieve and reap the benefits of all the 17 SDGs. Good nutrition comes with improved health and wellbeing (SDG3), enhanced educational and work productivity (SDGs 4 and 8), less poverty (SDG1), and reduced inequalities (SDGs 5 and 10). On the other hand, improved food security and nutrition will result in stronger and more sustainable environment, communities, and technologies (SDGs 6, 7, 9, 11–17). This Evidence Research Topic, *Nutrition and sustainable development goal 4: quality education*, is part of an innovative collection showcasing nutrition in the context of the SDGs. The Research Topic provides up-to-date evidence on the role and impact of nutrition in relation to educational outcomes, as well as a selection of activities that have been utilized to promote education and capacity building for youth and health workers.

Nutrition education plays an important role in supporting the acquisition of knowledge, skills and behaviors needed for healthy and sustainable food behaviors. Food literacy is defined as “*the scaffolding that empowers individuals, households, communities or nations to protect diet quality through change and strengthen dietary resilience over time. It is composed of a collection of inter-related knowledge, skills and behaviors required to plan, manage, select, prepare and eat food to meet needs and determine intake*” [(1), p. 54]. In this Research Topic, Groufh-Jacobsen and Medin present *Food literacy competencies in youth—a mini-review*. This review concluded there is a lack of agreement about effective measurement of youth food literacy and that current tools to measure food literacy in youth are inadequate. The authors urge continued effort to achieve consensus on how to measure food literacy.

Nutrition education can be utilized to support positive behavior change. In the study by Wakwoya et al., positive outcomes of an intensive nutrition education and counseling program in pregnant women in East Shoa Zone, Ethiopia are described. The authors describe an educational program including counseling sessions, take-home brochures (in local language) and short text messages, and how this was effective in reducing undernutrition and improving overall nutrition status among pregnant women. They propose this be part of regular antenatal care in this setting. Mekonnen et al., present an evaluation of a school-based pulse crop focused nutrition education strategy. Based on inadequate intake of protein-source foods, especially animal-origin foods, the authors evaluated effectiveness of pulses crop focused nutrition education in reducing thinness among school adolescent girls. The nutrition education did not significantly reduce magnitude of thinness, however, does provide an opportunity to consider further activities and that small, but positive changes in pulse crop consumption behavior may still occur.

It is important to understand the preferences of individuals when considering the development and delivery of nutrition education. Soam et al. highlights the importance of exploring dietary patterns to guide meal planning and preparation to reduce food waste. The authors assessed food preferences of individuals attending training programs at several higher education institutions in India, using a food and nutrition survey. The authors argue that there is a clear pattern in food preference modulated by age, gender, and region of residence/origin. Based on the findings the authors recommend on innovations in meal planning, based on preferences, to reduce food waste. In another study authored by Szczepanski et al., the authors explored 6th- and 10th-grade students' conceptions of the production of cow's milk to provide evidence for the development of effective teaching and learning opportunities. The study focused on the importance of students' conceptions to promote sustainable food production and consumption. It highlighted key factors that influence students' conceptualization and their significance while identifying varying degrees of maturity in students' conceptualizations. The authors recommend curriculum in the context of Education for Sustainable Development.

School is the common setting for formal educational activities. School food environments, school feeding programs, and/or nutrition education can impact school attendance, diet quality, nutritional status, and educational indicators. In the study by Mohammed et al., the authors report on the nutritional impact of the school feeding program in Ethiopia. The authors used BMI-for-age, measured by body mass index for age *z*-score (BAZ) and a food frequency questionnaire to develop a dietary diversity score. The authors recommend continued investment in the human capital development of children, and further studies to examine sustainability and long-term impact.

Education for sustainable development, economic growth, environment, and health has the potential to impact poverty and food and nutrition security, especially so in vulnerable children. Adeoya et al. outline a study that involved utilizing interactive teaching methods with grade 4 and 5 students to develop a disaster preparedness nutrition curriculum. The goal was the implementation of continuous nutrition education to empower children to make healthy food choices in daily life and reduce the risk of disaster-nutrition-related morbidity and mortality. In addition, Gajardo-Araya et al., describe how a higher level of general physical fitness, particularly cardiorespiratory fitness, plays an important role in mediating the influence of fatness on the academic achievement of Chilean adolescents. The authors conclude that physical fitness plays an important role in academic achievement and health and should be supported in educational settings.

Nutrition education and professional development for health professionals is important in moving toward achievement of SDG2, as these health cadres are often relied upon to deliver nutrition information. Fresán et al., discuss an example of assessing knowledge and attitudes regarding sustainable diets among health professionals in Spain. The authors identify the need for capacity building among Spanish health workers to promote healthy diets and call for greater efforts in training to build this capacity. This is similar to the findings from Ayande et al.'s study on the knowledge, attitudes, and practices of registered dietitians and nutritionists in Ghana, in the context of enteral and parenteral nutrition support. The authors found that there was limited training and exposure, impacting self-efficacy, and that there was a need to develop professional training programs for this audience.

The depth and diversity of the research included in this Research Topic highlights the value and potential of considering nutrition alongside Sustainable Development Goal 4: Quality education. It is clear that good nutrition can enhance academic outcomes, and that education is needed to enhance nutrition outcomes, underscoring the value of, and need to, consider these side-by-side. The evidence presented in this Research Topic highlights presence of gaps in terms of tools to measure food literacy and in our understanding of effective nutrition education, but that there are strategies that are being used successfully. For health workers, the need for nutrition literacy focused professional development programs has also been indicated to equip them for effective implementation of nutrition awareness activities.

## Author contributions

SB: Writing – original draft, Writing – review & editing. CB: Writing – original draft, Writing – review & editing. EM: Writing – original draft, Writing – review & editing. AD: Writing – original draft, Writing – review & editing. AM: Writing – original draft, Writing – review & editing.
